# A Novel Macrophage Subpopulation Conveys Increased Genetic Risk of Coronary Artery Disease

**DOI:** 10.1161/CIRCRESAHA.123.324172

**Published:** 2024-05-15

**Authors:** Jiahao Jiang, Thomas K. Hiron, Thomas A. Agbaedeng, Yashaswat Malhotra, Edward Drydale, James Bancroft, Esther Ng, Michael E. Reschen, Lucy J. Davison, Chris A. O’Callaghan

**Affiliations:** 1 Nuffield Department of Medicine, Wellcome Centre for Human Genetics (J.J., T.K.H., T.A.A., Y.M., E.D., J.B., L.J.D., C.A.O.), University of Oxford, United Kingdom.; 2 Nuffield Department of Orthopaedics, Kennedy Institute of Rheumatology, Rheumatology and Musculoskeletal Sciences (E.N.), University of Oxford, United Kingdom.; 3 Oxford University Hospitals NHS Foundation Trust, John Radcliffe Hospital, United Kingdom (M.E.R.).; 4 Department of Clinical Science and Services, Royal Veterinary College, Hatfield, United Kingdom (L.J.D.).

**Keywords:** atherosclerosis, coronary artery disease, cholesterol, LDL, genome-wide association study, macrophages, multiomics, single cell analysis

## Abstract

**BACKGROUND:**

Coronary artery disease (CAD), the leading cause of death worldwide, is influenced by both environmental and genetic factors. Although over 250 genetic risk loci have been identified through genome-wide association studies, the specific causal variants and their regulatory mechanisms are still largely unknown, particularly in disease-relevant cell types such as macrophages.

**METHODS:**

We utilized single-cell RNA-seq and single-cell multiomics approaches in primary human monocyte–derived macrophages to explore the transcriptional regulatory network involved in a critical pathogenic event of coronary atherosclerosis—the formation of lipid-laden foam cells. The relative genetic contribution to CAD was assessed by partitioning disease heritability across different macrophage subpopulations. Meta-analysis of single-cell RNA-seq data sets from 38 human atherosclerotic samples was conducted to provide high-resolution cross-referencing to macrophage subpopulations in vivo.

**RESULTS:**

We identified 18 782 cis-regulatory elements by jointly profiling the gene expression and chromatin accessibility of >5000 macrophages. Integration with CAD genome-wide association study data prioritized 121 CAD-related genetic variants and 56 candidate causal genes. We showed that CAD heritability was not uniformly distributed and was particularly enriched in the gene programs of a novel CD52-hi lipid-handling macrophage subpopulation. These CD52-hi macrophages displayed significantly less lipoprotein accumulation and were also found in human atherosclerotic plaques. We investigated the cis-regulatory effect of a risk variant rs10488763 on *FDX1*, implicating the recruitment of AP-1 and C/EBP-β in the causal mechanisms at this locus.

**CONCLUSIONS:**

Our results provide genetic evidence of the divergent roles of macrophage subsets in atherogenesis and highlight lipid-handling macrophages as a key subpopulation through which genetic variants operate to influence disease. These findings provide an unbiased framework for functional fine-mapping of genome-wide association study results using single-cell multiomics and offer new insights into the genotype-environment interactions underlying atherosclerotic disease.

**Novelty and Significance**

**What Is Known?**

- Macrophages play a central role in atherosclerosis and in the uptake of atherogenic lipids such as oxidized low-density lipoprotein cholesterol.
- Human atherosclerotic lesions contain multiple macrophage subpopulations, but the distinct contribution of the different subpopulations to the disease process is unclear.
- Genome-wide association studies have identified over 250 genetic risk loci for coronary artery disease (CAD), many of which might act specifically through macrophages.

**What New Information Does This Article Contribute?**

- High-resolution genome-wide mapping of CRE (cis-regulatory elements) generated from single-cell multiomics links 121 CAD risk variants to 56 candidate genes and uncovers CAD-relevant transcriptional networks in the macrophage response to oxidized low-density lipoprotein cholesterol.
- The genetic heritability of CAD varies substantially across macrophage subpopulations and is significantly enriched in a novel CD52-hi lipid-handling macrophage subpopulation.
- *FDX1* is a novel disease gene regulated by risk variant rs10488763 specifically in lipid-handling macrophages.

Macrophages have long been recognized as central players in atherosclerosis, but their involvement in the genetic component of CAD remains poorly understood. In this study, we constructed a high-resolution map of macrophage CREs, which allows linkage of 121 candidate risk variants at 44 genetic loci to their 56 target genes. Based on this CRE map, we systemically quantified the disease relevance across heterogenous macrophage subpopulations, and discovered that CAD heritability was particularly enriched in a novel CD52-hi lipid-handling macrophage subpopulation. Functionally, these macrophages displayed activated lipid metabolism pathways, accumulated less lipid upon oxidized low-density lipoprotein cholesterol exposure, and were present in human atherosclerotic plaques. Mechanistically, we identified a specific CAD risk variant, rs10488763, which was activated in these lipid-handling macrophages and, for the first time, implicated its target *FDX1* as a disease gene. Altogether, our work provides novel insights into the genetic underpinnings of CAD and macrophage heterogeneity, paving the way for future therapeutic intervention targeting specific macrophage subpopulations in atherosclerosis.

**In This Issue, see p 2**

**Meet the First Author, see p 3**

Coronary artery disease (CAD) is a leading global cause of morbidity and mortality^1^ and results from atherosclerosis, a chronic inflammatory process within the artery wall.^2^ Monocytes and macrophages play a central role in atherosclerosis and can engulf modified cholesterol-carrying lipoproteins to form lipid-laden foam cells within the lesions.^3–5^ Cells of this lineage can display remarkably plastic responses to their microenvironment,^6,7^ and human atherosclerotic lesions contain macrophages with proinflammatory M1-like to proresolution M2-like profiles.^8–10^ Although single-cell techniques have demonstrated heterogeneity in human plaque macrophage populations,^11–14^ the lesions profiled are typically in the late stages of atherogenesis, when phenotypes may be polarized by long-established factors including modified cholesterols, cellular necrosis, sustained immune cell activation, and concomitant cytokine release.^4^ The response of macrophages to cholesterol, its vectors, and modified derivatives is fundamental to atherosclerosis but has not been well defined at the single-cell level and may differ across macrophage subpopulations.^15,16^ Deeper insight into the responses of different macrophage subpopulations to atherogenic lipid could help to identify new potential therapeutic targets.

CAD has a large heritable component,^17^ and >250 genomic risk loci have been identified by large genome-wide association studies (GWAS).^18,19^ However, the majority of these variants are found in noncoding regions of the genome, often in linkage disequilibrium with multiple other variants.^20,21^ Despite considerable efforts,^22^ the cellular populations and the molecular mechanisms through which these genetic loci transmit disease risk are largely unknown. Recent advances in single-cell techniques provide new opportunities for investigating the function of noncoding variants by identifying cis-regulatory interactions in cellular subpopulations.^23–26^ This information can then be used to prioritize candidate causal genetic variants.^27–30^ Although it has been established that several CAD risk variants act specifically through macrophages,^31^ current single-cell studies have failed to address the genetic contribution of macrophages to the disease.^11,32,33^

The challenge in identifying such contributions is likely due to the limitations of using single-cell RNA-seq (scRNA-seq) or single-cell assay for transposase-accessible chromatin with high throughput sequencing (ATAC-seq) in isolation. The power of single-cell–transcriptomic-based approaches is heavily dependent on the accuracy of gene-enhancer mapping. Because these maps are typically constructed from bulk sequencing of immortalized cell lines or whole tissues, they do not fully capture gene regulation dynamics in disease contexts. Conversely, variants prioritized based on single-cell studies of chromatin accessibility alone have excess false positives, as only a subset of accessible chromatin sites have regulatory activity.^34^ We reason that joint single-cell profiling of both the transcriptome and chromatin accessibility directly in primary cells will bridge the gap between previous studies that only utilized 1 of the 2 modalities, providing a more comprehensive understanding of both the cellular and genetic components of human diseases.

In the current study, we undertake comprehensive single-cell RNA and multiomics profiling of human macrophages to characterize their transcriptomic and epigenomic response to atherogenic oxidized low-density lipoprotein cholesterol (ox-LDL). We construct a high-resolution map of macrophage CRE (cis-regulatory elements), enabling us to prioritize candidate causal CAD risk variants and partition CAD heritability across macrophage subpopulations. We identify a novel CD52-hi lipid-handling macrophage subpopulation that conveys more heritable risks of CAD and demonstrate the reduced lipoprotein accumulation in these cells. In addition, we illustrate how this approach provides mechanistic insight into the cis-regulatory effect of the CAD-risk single nucleotide polymorphism (SNP) rs10488763 at the *FDX1/RDX* locus. Our results provide an unbiased framework for functional fine-mapping of CAD GWAS results through partitioning of heritability among macrophage subpopulations and deliver the first genetic evidence for the diverse and divergent influences of different human macrophage subpopulations on atherosclerosis and its heritability.

## METHODS

### Data Availability

All sequencing data supporting the findings in this study have been deposited in the National Center for Biotechnology Information (NCBI) Sequence Read Archive under accession number PRJNA1102756. Processed data have been made available at the Zenodo repository with record ID 11001642. Computational analysis codes for reproducing results and figures presented in this study are available at https://github.com/jhjiang2020/multiome_paper.

See the Supplemental Material and the Major Resources Table for a detailed description of materials and methods.

## RESULTS

### Single-Cell Sequencing Identifies Cellular Heterogeneity in Human Monocyte-Derived Macrophages

**Figure 1.**
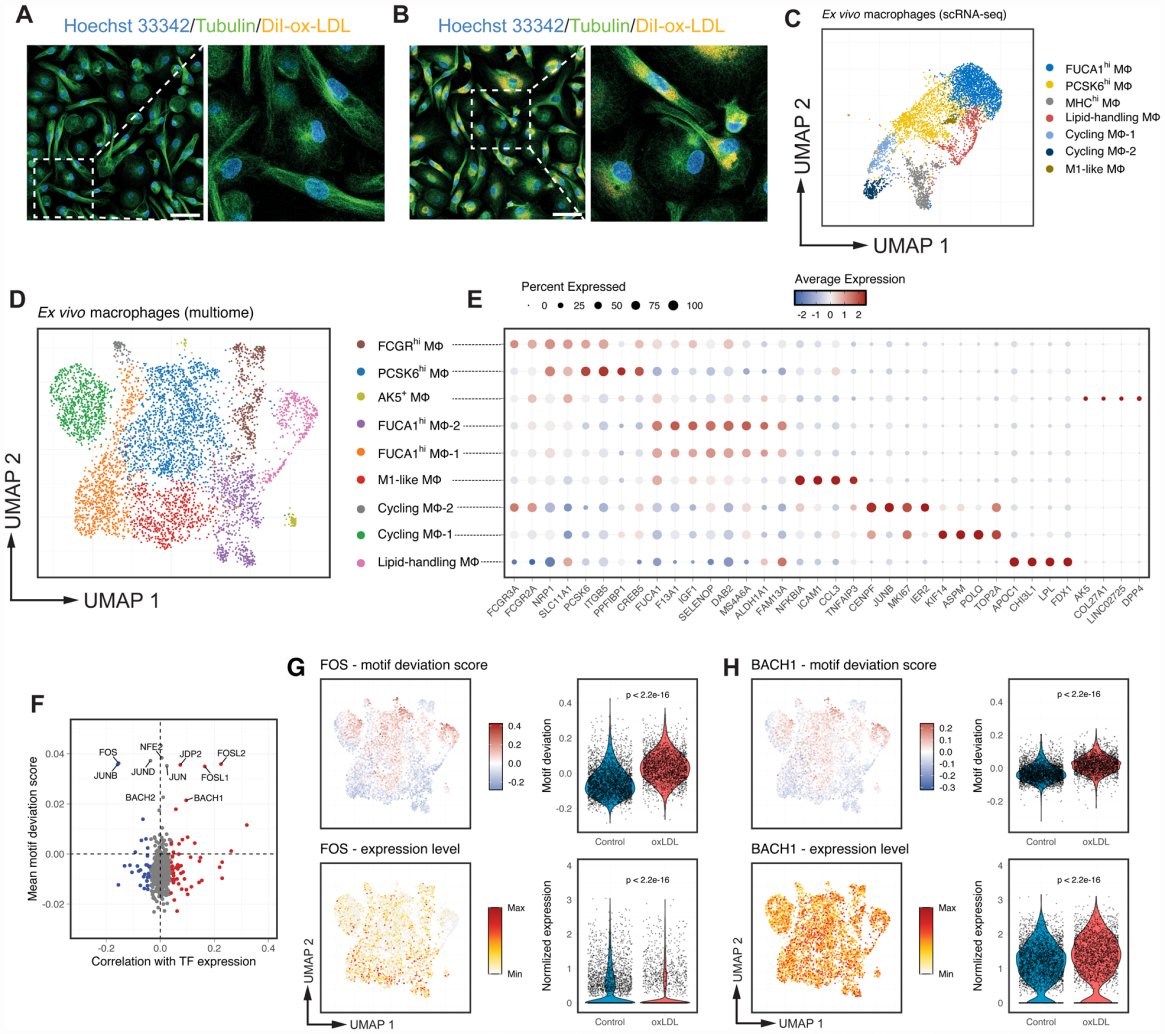
**Multiomics single-cell sequencing identifies cellular heterogeneity in human monocyte-derived macrophages and captures transcriptional regulators driving their response to oxidized low-density lipoprotein (ox-LDL) cholesterol. A** and **B**, Representative immunostaining of unexposed (**A**) and lipid-laden ex vivo macrophages (**B**), showing variations in cellular morphology and lipid accumulation. Scale bar: 50 µm. **C**, Uniform manifold approximation and projection (UMAP) visualization showing transcriptomically distinct macrophage subpopulations identified by single-cell RNA-sequence (scRNA-seq; n=4881 cells). **D**, Refined ex vivo macrophage subpopulations based on integrated single-nucleus RNA-seq and assay for transposase-accessible chromatin with high throughput sequencing (ATAC-seq) profiles and displayed as UMAP. **E**, Dot plot of the expression of selected marker genes for the 9 subpopulations in **D**; for each subpopulation, the color represents the average gene expression, normalized by sequencing depth and scaled across all cells; the size represents the percentage of cells expressing the gene. **F**, Mean TF (transcription factor) motif deviation scores (*y* axis, relative enrichment compared with background) plotted against their correlation with TF RNA expression (*x* axis, Pearson coefficient) in ox-LDL–treated cells. TFs are colored by significance (FDR <0.05) for negative (blue) or positive (red) correlation. **G**, Motif deviation scores (**top**) and normalized gene expression (**bottom**) for the AP-1 family TF *FOS*, shown on the UMAP and summarized by ox-LDL exposure status in the violin plot. Gene expression levels were min-max normalized for visualization purposes. **H**, Motif deviation scores (**top**) and normalized gene expression (**bottom**) for NFE2 family TF *BACH1*. *P* values were calculated using a Wilcoxon test. SNP indicates single nucleotide polymorphism.

Monocytes adhere to the vessel wall at sites of atherosclerotic lesions and are the predominant source of plaque macrophages.^3,4,35^ Primary human macrophages derived ex vivo from circulating monocytes are extensively used to study the molecular basis of atherosclerosis,^36–39^ and transform into a lipid-laden foam cell phenotype when exposed to ox-LDL. We observed significant heterogeneity in cell morphology among these macrophages (Figure 1A), and they exhibited various lipid-accumulation and distribution patterns after ox-LDL treatment (Figure 1B). To characterize the transcriptomic heterogeneity in macrophages and its impact on ox-LDL response, we first performed single-cell RNA sequencing in both unexposed and ox-LDL–exposed human macrophages (Figure S1A). After removing low-quality cells, we retained 4881 cells comprising 7 transcriptomically distinct clusters (Figure 1C). Consistent with our previous findings,^37,38^ exposure to ox-LDL significantly upregulated the expression of genes involved in lipid transport, such as *CD36*, *ABCA1*, and *ABCG1* (Figure S1B; Table S1).^40,41^ Kyoto Encyclopedia of Genes and Genomes pathway analysis demonstrated that ox-LDL triggered lysosomal activation and inhibited cholesterol biosynthesis (Figure S1C).

To better understand the epigenetic regulation of macrophage gene expression, we undertook single-cell multiomics, simultaneously characterizing the transcriptome and open chromatin profile of each cell, using single-nucleus RNA-seq and ATAC-seq, respectively (Figure S1A). The single-nucleus RNA-seq profiles were highly concordant with the scRNA-seq profiles (Figure S1D), and integration with the ATAC-seq open chromatin profile for each cell improved the resolution of the allocation of macrophage subpopulations. With this improved analysis, we identified 9 macrophage subpopulations (Figure 1D; Figure S1E and S1F; Table S2), including a cluster with high expression of lipid–metabolism genes (*APOC1* and *LPL*) even without ox-LDL exposure; therefore, we termed these lipid-handling macrophages (Figure 1D and 1E; Table S2).

Other subpopulations included an M1-like population showing high expression of proinflammatory cytokines (*CCL2*, *ICAM1*, and *CCL3*), 2 proliferating populations (*TOP2A*, *CENPA*, and *CENPE*), and a small AK5^+^ population (*ANKH*, *AK5*, and *MYO1B*) with some similarity in gene expression to osteoclasts.^42^ Subpopulations analogous to classic resting M0 macrophages included a PCSK6^hi^ population (*PCSK6*, *ITGB5*, and *SHC3*), 2 FUCA1^hi^ populations (*FUCA1*, *WWP1*, and *SELENOP*) and a transitional monocyte/macrophage FCGR^hi^ population (*FCGR3A*, *FCGR2A*, and *PCSK6*; Figure 1D and 1E).

### Single-Cell Multiomics Captures the Transcriptional Regulators Driving the Response to ox-LDL in Macrophages

We next assessed TF (transcription factor)-binding activity in individual cells by computing motif deviation scores, which represent motif-specific gain or loss of chromatin accessibility relative to the average cell profile (see Methods; Figure S1G). As TFs belonging to the same family usually share binding motifs, we examined the correlation between the motif deviation scores and the expression of each individual TF to understand the differential role of TFs within the same family. Using bulk approaches, we have previously identified enrichment of the binding motifs of AP-1 and NFE-2 factors in dynamic enhancer sites following exposure of human macrophages to ox-LDL.^38^ Both AP-1 and NFE-2 factors respond to oxidative stress.^43,44^ However, it is challenging to pinpoint the specific TFs involved in the ox-LDL response in macrophages, as they all share similar binding motifs. Here, with single-cell multiomics, we can now dissect the directional influence of individual AP-1 and NFE-2 family member TFs (Figure 1F). For example, the expression of 2 AP-1 family members *FOSL1* and *FOSL2* positively correlated with the chromatin accessibility of AP-1 motifs (Figure 1F), whereas another AP-1 factor *FOS* displayed a reverse correlation (Figure 1F and 1G). Expression of the NFE-2 family member *BACH1* was positively correlated with its motif binding (Figure 1F and 1H), but no significant correlation was observed for other NFE-2 family members.

### Analysis of Cis-Regulatory Elements Identifies Complex CAD-Associated Transcriptional Regulatory Networks

**Figure 2.**
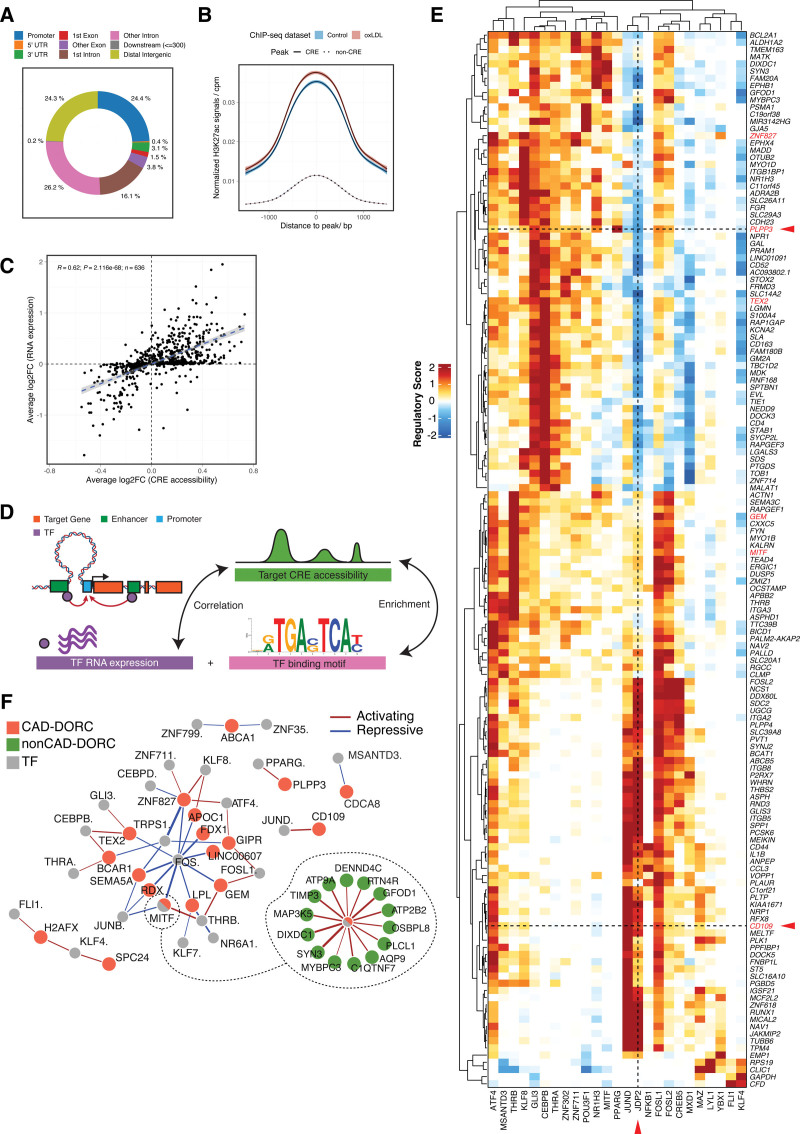
**Multiomics analysis identifies cis-regulatory elements and key transcriptional regulatory networks for coronary artery disease (CAD). A**, Location of CREs (cis-regulatory elements; n=22 555 CREs). **B**, Aggregated H3K27ac signals at CRE-peak and non-CRE-peak regions. Signals from unexposed (blue) and oxidized low-density lipoprotein cholesterol (ox-LDL)–exposed (red) human macrophages are plotted separately. Shaded regions indicate 95% CIs. **C**, Pearson correlation between ox-LDL–induced changes in CRE chromatin accessibility and in gene expression. n=636 DORC genes analyzed. **D**, Schematic diagram of the workflow for identification of transcription regulatory networks.^49^ The significance of each TF-gene interaction is tested by assessing whether the TF motif is enriched in the target CRE and whether the expression of the TF correlates with the accessibility of the target CRE (see Methods). **E**, Heatmap of regulation scores for the top 0.1% of TF (transcription factor)-DORC enrichments (n=25 TFs and n=147 DORCs). DORC genes are in rows and TFs are in columns. DORCs with CREs overlapping CAD-associated variants are marked with red. Positive and negative regulatory effects are shown in red and blue, respectively. Red arrows highlight the opposite regulatory effect of JDP2 on *CD109* and *PLPP3*. **F**, Network visualization of significant TF-DORC pairs for CAD-DORCs (absolute regulation score >1.5). Nodes represent CAD-DORCs (orange) and TF regulators (grey). Non-CAD-DORCs regulated by MITF are plotted separately (green discs). Red lines indicate activating effects, and blue lines indicate repressive effects. Line widths are weighted by the regulation score.

Single-cell multiomics data allowed us to identify cis-regulatory interactions by correlating chromatin accessibility at noncoding peaks with expression of nearby genes (<500 kb).^26^ We identified 18 782 unique CREs (cis-regulatory elements) spanning 6381 genes (Table S3). Of these CREs, 2642 were correlated with the expression of >1 gene, resulting in a total of 22 555 peak-gene pairs. The major locations of CREs were in introns (42.3%), promoters (24.4%), and intergenic regions (24.3%), with 42.6% of all CREs located >10 kb from any recognized transcription start site (Figure 2A).

To validate the predicted peak-gene interactions, we tested our macrophage CRE sets for enrichment against a wide range of functional annotations generated from public data sets. By incorporating blood expression quantitative trait loci (eQTL) data from GTEx^45^ and eQTLGen,^46^ we showed that eQTL SNPs were significantly overrepresented in the CREs (*P*=6.2×10^−10^; Figure S2A), with at least 1 eQTL SNP in 41.2% of CREs. The CREs were also enriched for activity-by-contact (ABC) super enhancers identified from enhancer-promoter contacts in mononuclear phagocytes^47^ (Figure S2B) and for genomic regions shown to interact with macrophage promoters by promoter capture Hi-C^48^ (Figure S2C).

In addition, we quantified the active enhancer signals over all open chromatin peaks using an H3K27ac ChIP-seq data set we generated in bulk macrophages.^38^ The average H3K27ac signal in CRE peaks was significantly higher than in non-CRE peaks (*P*<2.2×10^−16^) and exposure to ox-LDL increased the H3K27ac signals in CRE peaks (*P*=6.458×10^−6^) but not in non-CRE peaks (Figure 2B; Figure S2D).

We next applied the FigR framework^49^ to the multiomics data to understand the gene regulation dynamics in macrophages. To further reduce the background noise, we limited the downstream correlation analysis to a subset of 636 genes with a high density of CREs (≥9 CREs per gene; Figure S2E). These genes and their associated CREs constitute domains of regulatory chromatin (DORCs). Of these DORC genes, 529 (83.2%) were regulated by strong mononuclear cell ABC enhancers (ABC score >0.1; Figure S2F).^47^ Upon ox-LDL exposure, changes in chromatin accessibility at DORC CREs closely correlated with changes in the expression of DORC genes (R=0.62; *P*=2.1×10^−68^; Figure 2C).

For each DORC gene, we assessed their interactions with all detected TFs by 2 metrics: TF motif enrichment in CRE peaks, and the correlation between TF expression and CRE chromatin accessibility (Figure 2D). In total, we analyzed 588 300 TF-DORC pairs representing 925 TFs and 636 DORCs and identified 1612 strong interactions (|regulatory score| >1.5) involving 95 TFs and 526 DORCs (Table S4). The top 0.1% of TF regulators and their target DORCs with the maximum regulatory scores >2 are plotted in Figure 2E. Notably, several TFs, primarily in the AP-1 family, display opposite regulatory effects across different DORC genes (Figure 2E). For example, JDP2 has an activating effect on *CD109* but negatively regulates *PLPP3* (marked with arrows in Figure 2E).

Different DORCs that share similar TF associations constitute integrated gene regulatory networks. We collated all strong TF-DORC interactions (|regulatory score| >1.5) with CAD DORCs (DORC with CREs overlapping CAD-associated variants) and constructed a TF regulatory network for CAD in macrophages (Figure 2F). This network centered around members of the AP-1 family, with *FOS* and *JUNB* acting as repressors and *FOSL1*, *ATF4*, and *JUND* as activators. Other TF families, including CEBP and KLF, also targeted multiple CAD DORCs. The TF-DORC relationships can be complex; for example, one of the CAD DORC genes regulated by AP-1 factors includes *MITF*, which is itself a TF regulator and activates a wide range of downstream DORCs (Figure 2F).

### Macrophage CRE Map Prioritizes 121 CAD-Associated Variants Targeting 56 Genes

**Figure 3.**
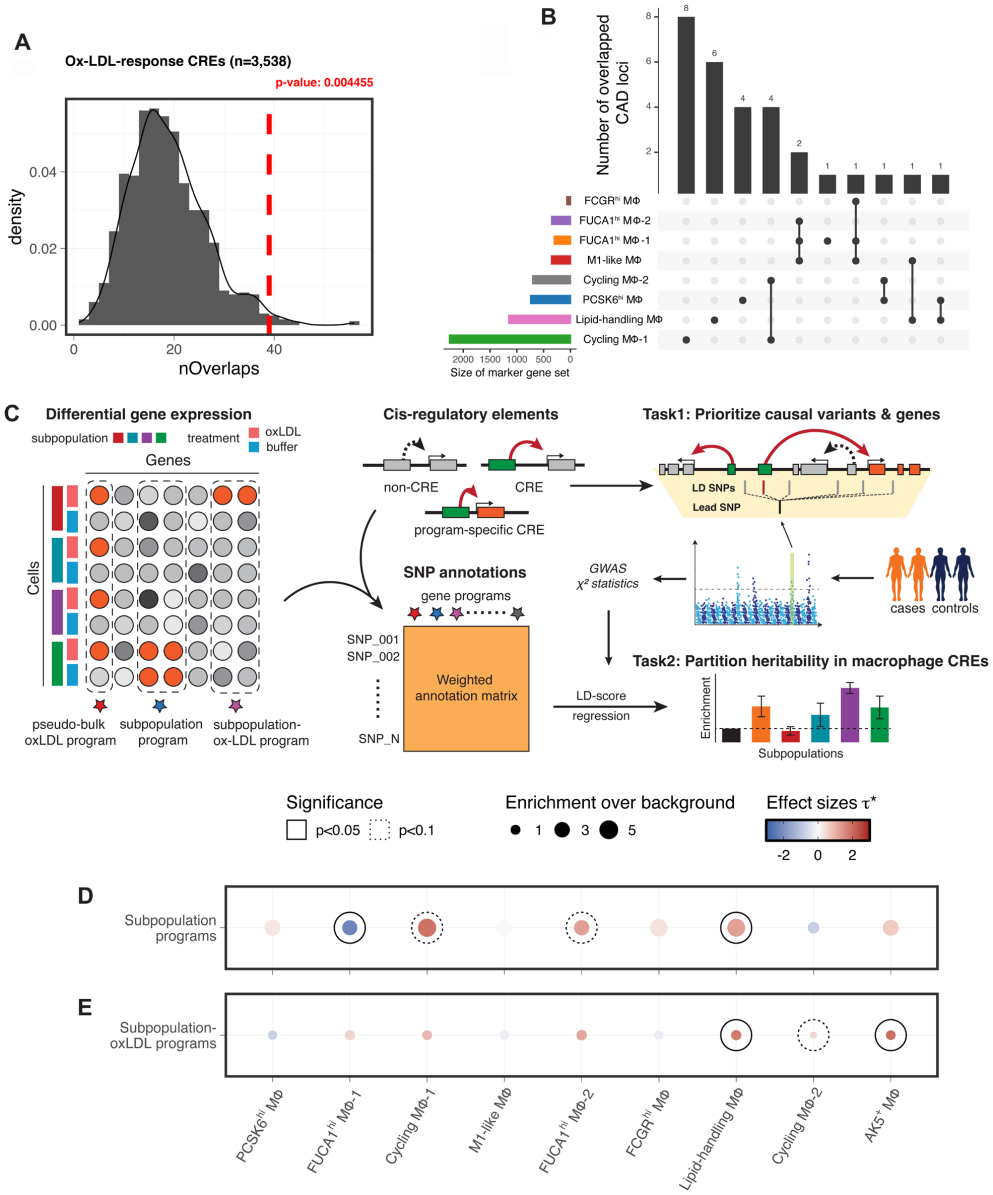
**Partitioning the genetic risk of coronary artery disease (CAD) prioritizes disease-critical macrophage subpopulations. A**, Histogram showing the null distribution of overlaps between CAD-associated SNPs and n=1000 sets of background peaks matched for GC% bias and average accessibility for all oxidized low-density lipoprotein cholesterol (ox-LDL)–response CREs (cis-regulatory elements). The observed overlap is marked by the red dashed line. **B**, Number of independent CAD loci mapped onto CREs targeting subpopulation-specific genes. Each set of subpopulation marker genes was binarily selected by a differential gene expression test using an FDR threshold of 0.05. Rows represent marker gene sets, and intersections between different sets are shown as connected dots. The size of each marker gene set is plotted to the left of the matrix, and the size of each intersection is plotted on the top. **C**, Genome-wide association studies (GWAS) heritability enrichment framework. **Left**, Deriving macrophage subpopulation gene programs, pseudo-bulk ox-LDL gene program, and subpopulation-ox-LDL gene programs from single-cell transcriptomic data. **Middle**, Converting gene programs into weighted genomic regions using the macrophage CRE map (**top**), then constructing single nucleotide polymorphism (SNP) annotation matrices by assigning the corresponding weights to 1000G SNPs overlapping each region (**bottom**). **Right**, After accounting for the linkage disequilibrium (LD) structure within GWAS risk loci, the putative causal variants and their target genes are prioritized by their overlaps with macrophage CREs (task 1); the relative enrichment of GWAS heritability for stratified subpopulation-specific/subpopulation-specific–ox-LDL–specific annotations are tested using the LD score regression model^51^ (task 2). **D** and **E**, Enrichments for CAD heritability in subpopulation gene programs (**D**) and subpopulation-ox-LDL gene programs (**E**). Dot size denotes the relative enrichment over background annotations. Color denotes the standardized effect sizes (see text) after conditioning on each other (**D**) or on a pseudo-bulk ox-LDL–response program (**E**) Nonzero τ* estimates reported at 2 significance levels (solid: *P*<0.05, dotted: *P*<0.1).

Noncoding disease-associated genetic variants often influence disease risk by altering the gene regulatory properties of CRE.^50^ The integration of CREs into the analysis of CAD GWAS results allows candidate causal variants to be linked to their target genes. Permutation-based testing demonstrated that macrophage CREs were enriched for CAD-associated variants—these CREs overlapped significantly more CAD-associated variants than comparable background control peaks did (*P*=0.00548; Figure S3A). The subset of CREs associated with ox-LDL–regulated genes (ox-LDL–response CREs) was also enriched for CAD-associated variants (*P*=0.004455; Figure 3A). We extended GWAS significant SNPs (adjusted *P*<5×10^−8^) to full summary statistics^18^ using stratified linkage disequilibrium score regression^51^ and showed that this enrichment was specific for CAD compared with nonrelevant traits (Figure S3B). These results strongly support the hypothesis that macrophages and their response to ox-LDL are causally involved in the heritability of CAD.

Of the 9516 variants in high linkage disequilibrium (r^2^>0.8 in the 1000G EUR panel) with the lead CAD variants in the NHGRI-EBI catalog, 121 SNPs were located within macrophage CREs and we linked these to 56 cis-regulated target genes (154 SNP-gene pairs; Table S5). In 101 pairs (71.4%), the SNPs and cis-regulated genes were >10 kb apart (median distance, 42.9 kb), and the cis-regulated gene was the nearest gene to the SNP only in 48 SNP-gene pairs (34.3%). These results demonstrate the limitations of traditional approaches, which prioritize variants by proximity. Furthermore, although 110 of the SNPs (90.9%) within macrophage CREs were eQTL SNPs,^45,46^ only 34.3% (53 of the 154 SNP-gene pairs) had the same target gene for both the CRE and the eQTL effect, suggesting that eQTL analysis alone will not identify the regulatory effects of many risk variants.

We benchmarked our macrophage CRE map against state-of-the-art ABC-enhancer mapping based on promoter-enhancer contact information from 35 public mononuclear cell data sets.^47^ Although macrophages are known for their involvement in atherosclerosis,^5^ we found no significant enrichment for CAD risk variants after annotating macrophage open chromatin regions with the ABC-enhancer map (61 950 peaks, targeting 19 969 genes; Figure S3C). We also found no significant enrichment for CAD risk variants in the subset of 9153 ABC-enhancer peaks targeting ox-LDL–response genes (Figure S3D). These results suggest that ABC-mapped mononuclear enhancers are limited in their power to prioritize causal CAD variants from background noise in primary macrophages.

Together, these findings demonstrate the value of using single-cell multiomics to generate a CRE map for the prioritization and interpretation of CAD risk variants. This focused approach provides greatly enhanced power and precision for investigating macrophage-specific CAD variants compared with traditional approaches.

### CAD Heritability Varies in Macrophage Subpopulations With Enrichment in Lipid-Handling Macrophages

As demonstrated above (Figure 1C through 1E), macrophage subpopulations differ in their transcriptomic and epigenetic profiles. We, therefore, reasoned that this would make them differ in the extent to which they are functionally influenced by CAD–risk variants and so the nature of their contribution to CAD heritability. More specifically, we hypothesized that the CAD heritability that is enriched in macrophage CREs (Figure S3A and S3B) could be further stratified to evaluate the genetic contribution of each macrophage subpopulation to the disease. In accordance with this hypothesis, of the 44 loci at which CAD SNPs overlap CREs, 29 of these loci overlap CREs whose target genes are only expressed in one or a few macrophage subpopulations (Figure 3B), indicating that their disease risk-modifying influence is likely to be specific to certain subpopulations. Because assayed DNA variants with genome-wide significance only explain a small portion of the genetic variance of CAD,^52^ we again extended the significant variants to all SNPs included in the GWAS summary statistics. For each macrophage subpopulation, we applied stratified linkage disequilibrium score regression to systematically assess the enrichment for genome-wide CAD heritability, rather than the enrichment for individual risk variants (Figure 3C).

In principle, the genetic contribution of macrophages can be divided into subpopulations based on their gene expression patterns, and the gene expression patterns reflect both the baseline and the ox-LDL-response signatures in the transcriptome. Using our previously published Pi strategy,^53^ we determined a series of weighted gene sets (gene programs) by scoring differential gene expression statistics for each subpopulation. A gene program characterizes either the baseline expression of a subpopulation (termed a subpopulation gene program) or the response of a subpopulation to ox-LDL (termed a subpopulation–ox-LDL gene program). As a comparator, we pooled all subpopulations and constructed a pseudo-bulk ox-LDL program characterizing the response of the whole bulk macrophage population to ox-LDL.

The gene programs were then converted into weighted genomic regions using our macrophage CRE map; all overlapping 1000G SNPs were assigned the corresponding weight for each region to construct the SNP annotation matrices required for heritability stratification. Heritability enrichment was defined as the proportion of SNP heritability explained divided by the proportion of SNPs included in each annotation matrix. As subpopulation-specific annotation matrices were weighted on a continuous scale, we also computed the standardized effect sizes τ*, defined as the proportional change in per-SNP heritability with a 1-SD increase in the SNP annotation weight, to assess the strength and statistical significance of heritability enrichment (see Methods).^27,54^

Compared with the background annotation constructed using all macrophage open chromatin regions, subpopulation gene programs show higher enrichment for CAD heritability (Figure 3D; Figure S3E). This is expected as regulatory elements have been widely reported to be enriched for disease and complex trait heritability.^51^ To test the independent contribution of each subpopulation gene program to the overall enrichment for CAD heritability, we further controlled subpopulation gene programs on each other using a multivariate linear regression model. The results showed that CAD heritability was relatively depleted (τ*=−2.24) in FUCA1^hi^ macrophages and remained significantly enriched (τ*=1.28) in lipid-handling macrophages (Figure 3D). This suggests that the baseline expression patterns in lipid-handling macrophages convey higher genetic risks for CAD with respect to other macrophage subpopulations.

Although different macrophage subpopulations had distinct subpopulation gene programs (Figure S3F), their subpopulation–ox-LDL gene programs were highly correlated, indicating a shared overall ox-LDL–response across macrophage subpopulations (Figure S3G). Therefore, instead of conditioning on each other, we used the pseudo-bulk ox-LDL gene program as the reference to compute the standardized effect size τ* for each subpopulation–ox-LDL program. For most subpopulations, τ* no longer deviated from zero (Figure 3E), meaning that the ox-LDL effect in these subpopulations is largely not subpopulation specific. However, there was still significant enrichment of heritability in the ox-LDL response in lipid-handling and AK5^+^ macrophages, with τ* of 1.81 and 1.97, respectively (Figure 3E).

Overall, our heritability analysis demonstrated that lipid-handling macrophages contribute more to the heritable component of CAD compared with other macrophage subpopulations, both in unexposed state and upon exposure to ox-LDL.

### Lipid-Handling Macrophages Display Activation of Genes and Transcription Regulators Involved in Lipid Processing and Clearance

**Figure 4.**
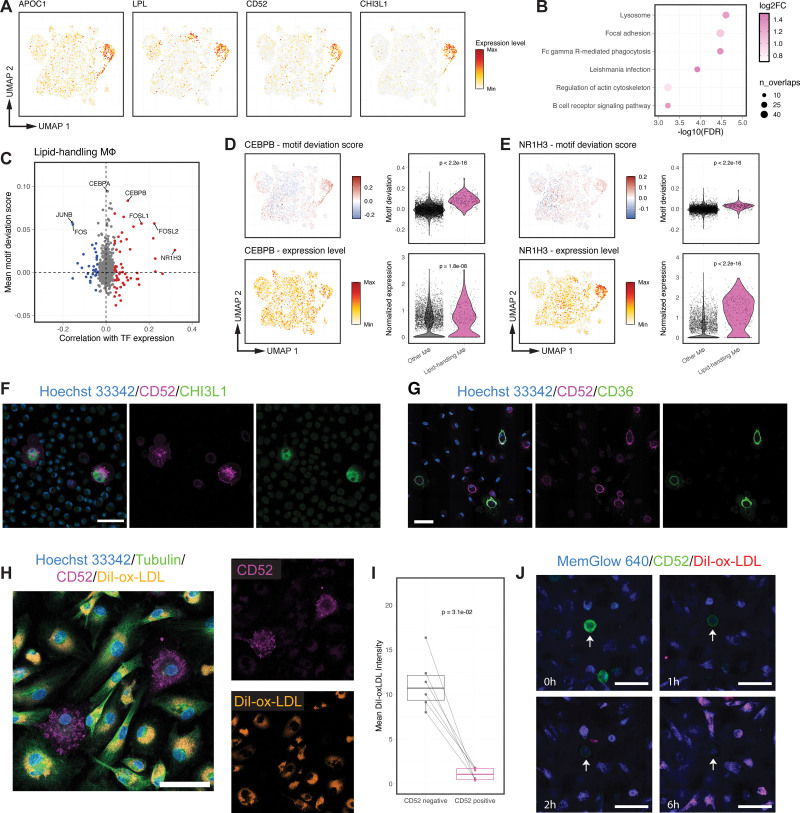
**Multiomics characterization of the lipid-handling macrophage subpopulation. A**, The expression of selected marker genes displayed on the uniform manifold approximation and projection (UMAP). Expression levels were min-max normalized for visualization purposes. **B**, Kyoto Encyclopedia of Genes and Genomes (KEGG) pathways enriched in the marker genes of lipid-handling macrophages. Color represents log2-fold change and size represents the number of overlapped genes in each pathway. **C**, Mean TF (transcription factor) motif deviation scores (*y* axis, the relative enrichment compared with background) compared with their correlation with TF RNA expression (*x* axis, Pearson coefficient) in lipid-handling macrophages. TFs are colored by significance (FDR<0.05) for negative (blue) or positive (red) correlation. **D**, Motif deviation scores (**top**) and RNA expression (bottom) for *CEBPB*. **E**, Motif deviation scores (**top**) and RNA expression (**bottom**) for *NR1H3*. **F** and **G**, Representative immunostaining of ex vivo macrophages for lipid-handling macrophage markers CD52, CHI3L1 (**F**), and CD36 (**G**). **H**, Representative immunostaining of ex vivo macrophages for lipid accumulation after 48 hours treatment with DiI-oxidized low-density lipoprotein cholesterol (ox-LDL). **I**, Quantification of the intracellular level of DiI-ox-LDL in **H**, shown as average fluorescence intensities (arbitrary units) per biological replicate (n=6). **J**, Representative live-cell imaging of ex vivo macrophages for lipid accumulation at different time points post-DiI-ox-LDL exposure, with a CD52^+^ cell marked by the white arrow. Cells were briefly incubated with Fc-muted CD52 antibody for 1 hour before the assay to minimize the effect of antibody binding. Scale bar: 50 µm. *P* values were calculated using a Wilcoxon test in **D** and **E**, and a paired Wilcoxon test in **I**.

Having identified a dominant role for lipid-handling macrophages in the heritability of CAD, we sought to further characterize this macrophage subpopulation. Both scRNA-seq and single-nucleus RNA-seq data revealed a significantly increased expression of apolipoprotein *APOC1* and lipoprotein lipase *LPL* in these cells (Figure 4A; Figure S4A), both of which are involved in lipoprotein metabolism.^55^ Kyoto Encyclopedia of Genes and Genomes and Gene Ontology Biological Process enrichment analyses suggested an enhancement in phagocytosis (Figure 4B) and fatty acid metabolism (Figure S4B) within these cells. The membrane glycoprotein *CD52* and nonenzymatic chitinase-3 like-protein-1 *CHI3L1* were also uniquely expressed in lipid-handling macrophages (Figure 4A; Figure S4A), while their roles in macrophage lipid metabolism, if any, are uncharacterized.

Notably, the marker genes of lipid-handling macrophages (133 genes with FDR <0.05 and log2FC >1; Table S1) were significantly overrepresented in a gene regulatory network associated with subcutaneous fat and liver (GRN51, FDR=1.42×10^−20^; Figure S4C).^56^ The expression of this network strongly correlates with body mass index and blood triglyceride levels in a CAD patient cohort (Figure S4D).

We then identified the transcriptional regulators specific to lipid-handling macrophages. CEBP family TFs showed the largest motif deviation score for these macrophages (Figure 4C), with *CEBPB* expression correlating positively with CEBP–motif binding activity (Figure 4C and 4D). A strong correlation was also observed between expression and motif usage for *NR1H3*, a key transcriptional regulator of genes involved in fatty acid metabolism (Figure 4C and 4E).^57^

To functionally validate these findings, we first used immunofluorescence to confirm key marker gene expression and observed coexpression of *CD52*, *CHI3L1*, and the well-documented ox-LDL uptake receptor *CD36*^58^ in unexposed macrophages (Figure 4F and 4G; Figure S4E and S4F). Next, we performed a lipid uptake assay using DiI-labeled ox-LDL, which demonstrated reduced accumulation of intracellular ox-LDL in CD52^+^ macrophages compared with CD52^−^ macrophages (Figure 4H and 4I). This reduced lipid accumulation was further supported by live-cell imaging, as early as 2 hours post ox-LDL exposure (Figure 4J; Video S1). After ox-LDL treatment, lipid-handling macrophages exhibited increased expression of *CD36* (Figure S4G), a trend also seen in other subpopulations. The upregulation of *CD36* even in the unexposed state would be expected to increase ox-LDL uptake, so the reduced ox-LDL accumulation observed is consistent with CD52^+^ lipid-handling macrophages having more efficient lipid/cholesterol processing and clearance capacities.

### Meta-Analysis of 38 Human Atherosclerotic Samples Enables High-Resolution Mapping of Macrophages in Plaques

Most macrophages in atherosclerotic lesions are derived from circulating monocytes^3,35,59^ as were the macrophages we profiled; as plaque macrophages are exposed to multiple atherogenic influences, we sought to determine whether the CD52-hi lipid-handling macrophage subpopulation is a reproducible subpopulation in human atherosclerotic plaque.

We started with a meta-analysis of scRNA-seq data from 38 human coronary and carotid artery plaque samples from 5 independent studies^12–14,60,61^ (Figure S5; Supplemental Notes). After read alignment, sample QC, and doublet removal, we retained a total of 126 685 cells across 8 broad cell types (Figure S6A and S6B). We then focused on myeloid cells (n=20 458) based on expression of *CD14*, *CD68*, and *AIF1*.

**Figure 5.**
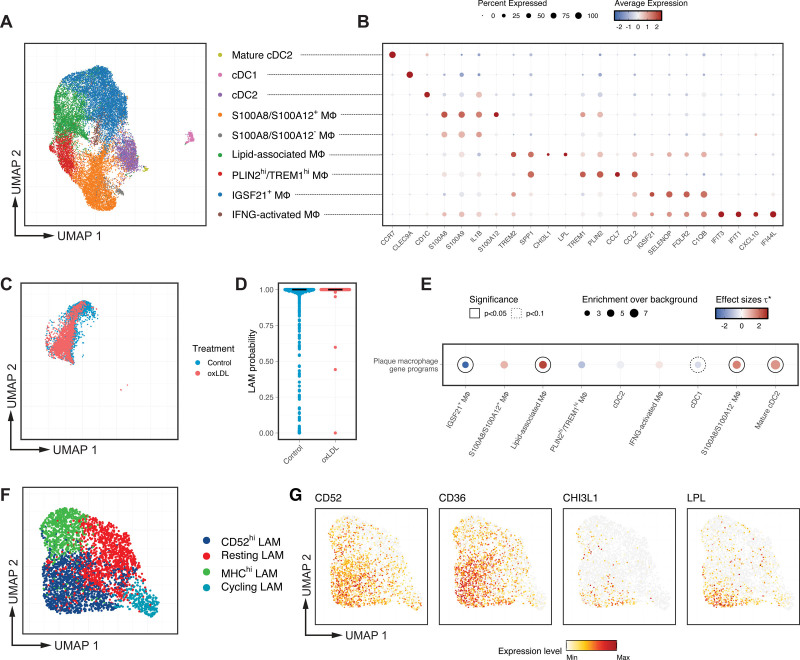
**Meta-analysis of 38 human plaque samples enables high-resolution mapping of plaque macrophages in vivo. A**, Uniform manifold approximation and projection (UMAP) visualization of the plaque macrophage atlas: multiple subpopulations of plaque macrophages demonstrated by meta-analysis of 38 samples from 5 independent studies.^12–14,60,61^ **B**, The expression of selected marker genes for subpopulations in **A** is summarized in the dot plot, where the color represents the average gene expression, normalized by sequencing depth and scaled across all cells; the dot size represents the percentage of cells expressing the gene in the subpopulation. **C**, Single-cell transcriptomic profiles of ex vivo macrophages projected onto the plaque macrophage atlas (**A**) colored by treatment, demonstrating close mapping to the lipid-associated macrophage (LAM) subpopulation in plaques. **D**, Predicted probabilities of ex vivo macrophages being classified as plaque LAM, shown as individual data points along with the median, colored by treatment. **E**, Enrichments for coronary artery disease (CAD) heritability in subpopulation gene programs of plaque macrophages. Dot size denotes the relative enrichment over all macrophage open chromatin regions. Color denotes the standardized effect sizes after conditioning on each other. Nonzero τ* estimates reported at 2 significance levels (solid: *P*<0.05, dotted: *P*<0.1). **F**, UMAP visualization of plaque LAMs in **A** but clustered at a higher resolution. **G**, The expression of selected lipid-handling macrophage markers displayed on the UMAP in **F**. Expression levels were minimum-maximum normalized for visualization purposes.

Next, we reintegrated the scRNA-seq data to replicate the macrophage subpopulations reported in previous studies.^61–63^ In total, we identified 6 heterogeneous macrophage clusters (Figure 5A and 5B; Figure S6C) including 2 S100A8^hi^ clusters S100A8/S100A12^+^ and S100A8/S100A12^−^ macrophages, an IFNG-activated cluster, an IGSF21^+^ cluster and 2 TREM2^+^ clusters expressing marker genes associated with lipid metabolism (*SPP1*, *CD9*, and *FABP5*). We termed these 2 clusters lipid-associated macrophages (LAMs) and PLIN2^hi^/TREM1^hi^ macrophages for consistency with previous reports.^61,64^ We also identified 3 previously reported clusters for dendritic cells, termed cDC1, cDC2, and mature cDC2.^61^

To investigate how monocyte-derived macrophages map to in vivo plaque macrophages, we conducted an integrative analysis using scRNA-seq profiles from our ex vivo macrophages and our plaque macrophage atlas. To our surprise, most ex vivo macrophages, both unexposed and exposed to ox-LDL, mapped closely to the TREM2^+^ LAM population in plaques (Figure 5C). This transcriptomic similarity was further supported by correlation analysis of the pseudo-bulk profiles (Figure S6D). In addition, we trained a machine-learning macrophage classifier with a deep generative model^65^ on the plaque macrophage atlas, and almost all ex vivo macrophages were predicted to be LAM with high confidence (Figure 5D). Importantly, we also expanded our heritability analysis to plaque macrophage populations and found that LAM, like ex vivo macrophages, showed a strong enrichment for CAD heritability (Figure 5E).

### CD52-hi Subpopulation Expressing High Levels of Lipid-Processing Genes Is Also Found Among Plaque Lipid-Associated Macrophages

We have demonstrated the transcriptomic, epigenomic, and functional heterogeneity among ex vivo macrophages. Because they robustly mapped to the LAM population in plaques, we hypothesized that plaque LAMs would also be heterogeneous with functionally distinct subpopulations. We anticipated that the increased sample size of our plaque macrophage atlas would allow us to discern macrophage heterogeneity at a much higher resolution than previously achieved and so discover rare populations not characterized in previous studies.

To ensure the robustness and reproducibility of newly identified macrophage subpopulations, we performed cluster stability analysis, testing a wide range of clustering parameters after correcting for batch effects (Figure S5; Methods and Supplemental Notes). As a result, we identified 4 transcriptomically distinct subpopulations within plaque LAMs (n=3645; Figure 5F; Figure S6E) in both coronary and carotid artery samples (Figure S6F). These LAM subpopulations broadly shared the gene expression patterns we had identified in various ex vivo subpopulations (Figure S6G and S6H). Notably, a subset of CD52-hi LAM displayed strong expression for genes involved in lipid processing and metabolism (*LPL*, *CD36*, and *PPARG*; Figure 5G; Figure S6G). Although CD52 was expressed in >1 plaque macrophage subpopulation, the coexpression of *CD52*, *CHI3L1*, and *LPL* was only observed in TREM2^+^ LAMs (Figure S6I). This is consistent with the lipid-handling phenotype we identified in ex vivo macrophages being a unique feature specific to LAMs.

### Integrative Analysis Reveals the Regulatory Mechanisms of CAD Risk Loci Targeting *MITF* and *LPL*

Given the finding of plaque macrophages with the lipid-handling phenotype and the strong enrichment for CAD heritability in lipid-handling macrophage gene programs, we next sought to investigate the CAD risk variants affecting this subpopulation and their regulatory mechanisms. Among the variants conveying CAD heritability, 19 variants (at 6 independent risk loci) with genome-wide significance were mapped to the marker genes of lipid-handling macrophages (Figure 3B). These risk variants were present at CREs targeting *MITF*, *LPL*, *FDX1/RDX*, *BCAR1*, *TEX2*, and *PLPP3*. We have previously characterized the regulatory mechanism of one such variant in the *PLPP3* gene based on studies of bulk macrophage populations,^38^ providing important orthogonal independent validation of the current approach.

**Figure 6.**
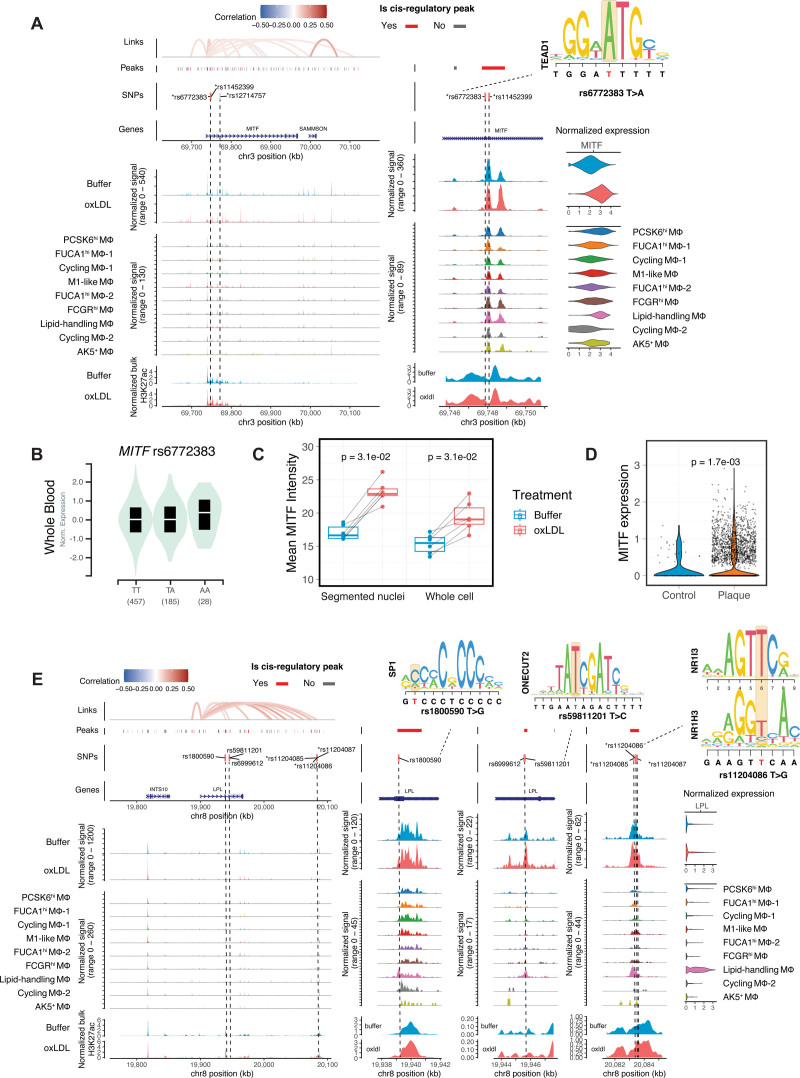
**Chromatin regulatory landscapes of coronary artery disease (CAD) risk variants in lipid-handling macrophages. A**, Genomic plots for the region encoding CAD risk variants rs6772383 and rs11452399, which localize to a CRE (cis-regulatory element) at the *MITF* locus. Tracks (from **top** to **bottom**) showing cis-regulatory interactions (links), ATAC-seq peaks (peaks), CAD-associated variants (SNPs), genomic annotations (genes), ATAC-seq signals aggregated by treatment, ATAC-seq signals aggregated by subpopulations, enhancer signals from H3K27ac ChIP-seq. Higher resolution coverage plots centered on the cis-regulatory peak are plotted in the **middle**. Violin plots of aggregated gene expression are shown to the **right**. A red bar indicates significant CREs, and eQTL SNPs are marked with an asterisk. **B**, Violin plot showing the allele-specific effect of rs6772383 on *MITF* expression in human blood based on GTEx data. **C**, Quantification of the immunofluorescence staining of ex vivo macrophages for MITF, shown as average nuclear and intracellular fluorescence intensities (arbitrary units) per biological replicate (n=6). **D**, *MITF* expression in lipid-associated macrophages (LAMs) from plaques vs LAMs from control unaffected arteries. **E**, Genomic plot similar to that in **A**, but showing CAD risk variants rs1800590, rs59811201, rs11204085, rs11204086, and rs11204087 overlapping CREs at the *LPL* locus. *P* values were calculated using a paired Wilcoxon test in **C**, and a Wilcoxon test in **D**.

*MITF* is a key TF influencing lysosome function and autophagy.^66,67^ An intronic variant rs12714757 has been associated with CAD risk in both European and East Asian populations (β=0.033 and 0.048, respectively).^18,68^ In our analysis, 2 closely linked SNPs rs6772383 and rs11452399 overlapped an intronic CRE of *MITF* (Figure 6A). At rs6772383, the risk T allele disrupts a TEAD1-binding site and is associated with decreased *MITF* expression in GTEx whole blood eQTL data (Figure 6A and 6B; Figure S7A), indicating that *MITF* expression is protective against atherosclerosis. Exposure of cells to ox-LDL resulted in a moderate increase in enhancer histone signals flanking this CRE (Figure 6A) and highly significant increases in both the chromatin accessibility of this CRE (*P*=3.3×10^−8^) and *MITF* expression (average log2FC=0.39; *P*<2.2×10^−16^). Ox-LDL–induced expression of *MITF* was confirmed at the protein level using immunofluorescence staining (Figure 6C; Figure S7B), and a similar trend was observed using Western blot analysis (Figure S7C and S7D). In plaque macrophages, *MITF* was predominantly expressed in the LAM subpopulation (Figure S7E), and its expression was upregulated in LAMs from disease tissue as compared with adjacent control tissue (Figure 6D).

The *LPL* gene encodes lipoprotein lipase, which plays a role in lipoprotein uptake and is one of the strongest marker genes of lipid-handling macrophages (Figure 4A); its expression is increased by ox-LDL exposure (average log2FC=0.32; *P*=3×10^−4^). We identified 6 CAD risk variants in 3 different CREs at the *LPL* locus (Figure 6E): rs1800590 in a 5′ untranslated region (UTR) CRE, rs6999612 and rs59811201 in an intronic CRE, and rs11204085/6/7 clustered in a distal intergenic CRE (β=0.091, 0.126, and 0.028 in European population for these 3 loci, respectively). Although no significant accessibility change was found in any of the 3 CREs with ox-LDL exposure (likely because they were already highly accessible), enhancer histone signals increased 70.9% in the CRE containing rs11204086 and 43.0% in CRE containing rs1800590. The protective G allele of rs11204086 disrupts a binding motif for NR1H3 (Figure 6E) and is associated with higher blood expression of *LPL* in eQTL data (Figure S7F). NR1H3 itself is activated notably in lipid-handling macrophages (Figure 4E) indicating that the cis-regulatory effect of rs11204086 may be mediated by a subpopulation-specific transcriptional network.

### CAD-Associated Variant rs10488763 Alters Chromatin Structure at an AP-1 Site and Regulation of *FDX1* Expression by CEBPB

**Figure 7.**
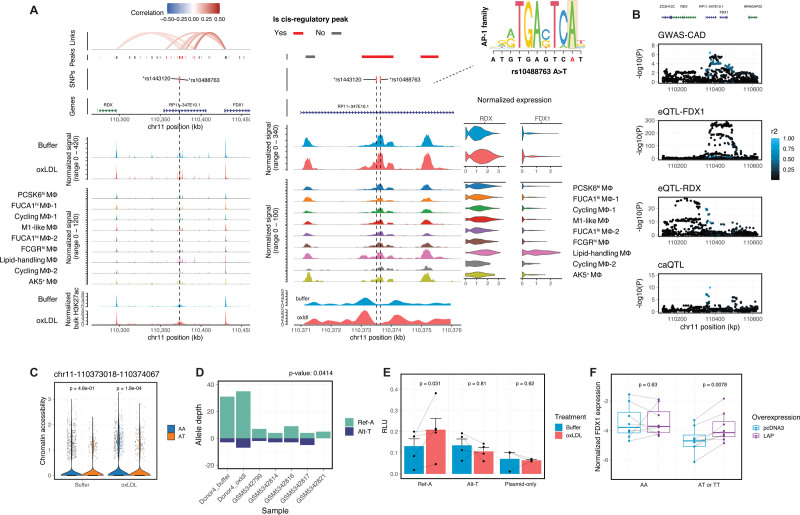
**Coronary artery disease (CAD) risk variants at the cis-regulatory region of *FDX1* and *RDX*. A**, Genomic plots of CAD-associated genome-wide association studies (GWAS) variant rs10488763 at the FDX1/RDX locus, similar to the plots shown in Figure 6A. **B**, Association of rs10488763 variant with CAD (GWAS-CAD), *FDX1* expression (eQTL-FDX1), *RDX* expression (eQTL-RDX, note smaller scale of *y* axis), and chromatin accessibility (caQTL) at the *FDX1/RDX* locus. SNPs are colored by their LD with rs10488763. **C**, Chromatin accessibility of the cis-regulatory peak in **A** grouped by genotype from multiomics data (n=3 for A/A, n=1 for A/T). **D**, Allelic sequencing coverage of rs10488763 in 7 heterozygous samples, showing higher accessibility in the chromosome containing the protective A allele compared with the T allele. Reads are aggregated from snATAC-seq data sets of primary human macrophages and human plaques.^70^ Sequencing coverage on the alternative allele shown in negative values for visualization purposes. **E**, Dual luciferase reporter assays in primary human macrophages with and without ox-LDL treatment testing the allelic enhancer activity of rs10488763 (n=5 biological replicates). Signals normalized to Renilla luciferase activities. **F**, RT-qPCR measurement of *FDX1* expression. Primary macrophages (n=8, 6, 2 for AA, AT, and TT samples, respectively) were transfected with LAP (liver-enriched activator protein) overexpression plasmid or control pcDNA3 plasmid. *GAPDH* was used as the reference gene. *P* values were calculated using a Wilcoxon test in **C** and a paired Wilcoxon test in **D**, **E**, and **F**.

From the CRE-based variant prioritization analysis, we identified 2 eQTL SNPs (rs10488763 and rs1443120) in tight linkage disequilibrium (r^2^=1 in EUR panel) that overlap a distal CRE for *FDX1* and *RDX*. This CRE is 56 and 77 kb upstream of the transcriptional start sites of *FDX1* and *RDX*, respectively (Figure 7A). The alternative alleles of these 2 SNPs were associated with higher CAD risk (β=0.036)^18,68^ and lower expression of both *FDX1* and *RDX* in whole blood (Figure S8A). However, CAD summary statistics and eQTL mapping suggest that the GWAS signal aligns most clearly with the eQTL signal of *FDX1* rather than of *RDX* (Figure 7B; Figure S8B). We also found elevated *FDX1* in plaque LAMs (*P*=7.7×10^−5^; Figure S8C) compared with nonplaque tissue LAMs, while *RDX* expression remained unchanged (Figure S8D). These results indicate that *FDX1* is the likely causal gene influenced by one or both CAD variants rs10488763 and rs1443120 at this locus.

As the risk T allele of rs10488763 perturbs the binding motif for AP-1 factors (Figure 7A), we hypothesized that this SNP exerts a cis-regulatory effect by modulating local chromatin structure. The rs10488763 SNP has been linked to a chromatin accessibility QTL locus (caQTL) in iPSC-derived macrophages in an infection model^69^ (Figure 7B; Figure S8B). Consistent with this caQTL signal, we found higher chromatin accessibility in ox-LDL–treated macrophages that are homozygous for the protective A allele, compared with those that are heterozygous for this allele (*P*=1.8×10^−4^; Figure 7C). We further compared the single-nucleus ATAC-seq (snATAC-seq) signal of rs10488763 in heterozygous donors and found that on the chromosome containing the protective A allele, this site was more accessible in both ex vivo macrophages and in human plaque cells^70^ (*P*=0.041; Figure 7D).

In addition to the chromatin modulating effect of rs10488763, chromatin accessibility at the locus of this variant is also increased by ox-LDL (*P*=0.015; Figure 7A), and this is associated with a 45.3% increase in the local H3K27ac enhancer signal (Figure 7A, bottom). To test whether rs10488763 alleles differentially affect this ox-LDL–induced enhancer activation, we transfected primary macrophages with luciferase reporter plasmids containing each allele. We observed a significant allelic difference, with ox-LDL triggering increased enhancer activity with the protective A allele (average 1.58-fold, *P*=0.031) but not at all with the risk T allele (Figure 7E).

Inspection of our CEBPB ChIP-seq data from bulk macrophages^38^ revealed a CEBPB-binding site 300 bp downstream of the rs10488763 SNP within the same CRE (Figure S8E). Overexpression of the activator isoform of CEBPB (LAP [liver-enriched activator protein]) induced a 7-fold increase in the enhancer activity of this CRE (Figure S8F). To test whether the cis-regulatory effect of rs10488763 is mediated by the recruitment of CEBPB, we overexpressed LAP in primary macrophages and quantified the expression of *FDX1* using RT-qPCR (Figure 7F; Figure S8G). Consistent with the eQTL signals in whole blood samples (Figure 7B), *FDX1* expression was significantly lower in ex vivo macrophages carrying the risk T allele (*P*=0.0039; Figure 7F). Overexpression of CEBPB compensated for this reduction of *FDX1* expression in macrophages carrying the T allele (*P*=0.016; Figure 7F), while having no additional effect in macrophages homozygous for the A allele (*P*=0.63; Figure 7F).

Together, our analyses link the GWAS signal at the rs10488763 locus to *FDX1* and demonstrate an allelic effect on the ox-LDL–induced enhancer activation and change in chromatin accessibility. Our data are consistent with a model in which the interaction of AP-1 TFs is altered by allelic variation at this SNP, which influences CEBPB recruitment to the CRE and so upregulation of *FDX1* (Figure S9). Given that *FDX1* and *CEBPB* were specifically expressed in lipid-handling macrophages, our results highlight the crucial role of lipid-handling macrophages as the causal subpopulation with an active transcriptional regulatory network required for the CAD risk variant rs10488763 to take effect.

## DISCUSSION

### Beyond Current Single-Cell Studies on Plaque Cells

Large-scale genome-wide association studies have identified many genomic loci associated with CAD, but the molecular mechanisms accounting for these associations are largely unknown. Recent studies using single-cell sequencing approaches on atherosclerotic lesions have provided insights into how these risk loci affect specific cell types such as endothelial and smooth muscle cells, yet failed to capture the genetic contribution of macrophages.^11,30,32,33^ The number of cells available from lesions is typically limited, and most are sampled from advanced lesions. In these lesions, the functional pathogenic activity of environmentally derived stimuli, such as atherogenic lipids, may be historic, making genotype-environment interactions difficult to evaluate. For macrophages, these limitations are especially problematic as macrophages are highly plastic and gene regulatory programs are strongly influenced by prevailing stimuli and neighboring cellular activity.^69,71^ To overcome this, we have studied primary human macrophages derived ex vivo from circulating monocytes, a major source of plaque macrophages in vivo.^15,35^ These ex vivo macrophages allow us to characterize the transcriptional regulatory dynamics before and after exposure to the atherogenic lipid, ox-LDL, and to comprehensively evaluate the contributions of different macrophage subpopulations to CAD heritability.

### CAD Variant Prioritization in Macrophages

The analysis of CREs offers a powerful route to further understand CAD heritability. The macrophage CREs that we identified account for 11.5% of the total SNP heritability for CAD and contain 121 risk variants with genome-wide significance. The advantages of the CRE approach over eQTL analysis are evident from the small fraction of these 121 variants that have a cis-regulatory link to the gene with which they are associated by eQTL analysis. Indeed, multiple studies have now reported limited overlaps between eQTLs and GWAS trait-associated variants,^72–74^ which highlights the need for other approaches to identify causal genes.^75^

Single-cell sequencing approaches circumvent the need for prior identification and segregation of disease-relevant cell types for functional genomic studies.^76^ Most single-cell variant prioritization studies integrating single-cell epigenome data only use open chromatin regions, but such regions are not necessarily regulatory.^30,33^ Only 6.9% of open chromatin peaks have cis-regulatory effects in our macrophage data, so colocalization analysis using all open chromatin sites would likely include many false positives for causal variants. A further issue is that the presence of many nonregulatory peaks in accessible chromatin regions drowns out enrichment signals from true regulatory elements, leading to false-negative results in the prioritization of causal cell types or cell states. This is evident in macrophages for which we have previously profiled open chromatin regions in bulk samples and found no global enrichment for CAD risk loci.^38^ Similar false negatives were also observed in single-cell ATAC-seq studies on human plaque and heart macrophages.^30,33^

In this study, we have developed a computational workflow to partition SNP heritability among macrophage subpopulations. Our results demonstrate that CAD heritability is not uniformly distributed across these subpopulations. CAD-associated variants likely act by altering gene regulation, so different macrophage subpopulations with distinct transcriptomes and epigenetic profiles are likely to be differently affected by these causal variants. Therefore, the impact of a particular CAD-associated variant may differ between subpopulations. In particular, we show that CAD heritability is enriched in the marker gene program and the ox-LDL response gene program specific to a subset of macrophages, namely the lipid-handling macrophages. We have provided further orthogonal validation of the relevance of such subpopulation-specific CAD variants at the *MITF*, *LPL*, and *FDX1* loci.

### Functional Characterization of Lipid-Handling Macrophages

The lipid-handling macrophage subpopulation is distinguished by its activation of lipid-processing genes and transcriptional networks driven by CEBP and NR1H3 family factors. This subpopulation can be tracked in vitro through immunofluorescence staining of a membrane glycoprotein CD52. These CD52-hi macrophages exhibit a higher expression of ox-LDL receptor CD36, yet surprisingly, accumulate much less ox-LDL than other subpopulations. In addition, we noted a variation in the proportion of CD52-hi cells, ranging from 1% to 10% across different biological donors. Although in the current study we only recruited healthy donors, this can be clinically relevant considering the significant lipid-clearing capacities these cells possess.

Through a comprehensive meta-analysis of 38 human atherosclerotic samples from 5 independent studies, we constructed by far the largest single-cell atlas for plaque macrophages with a total of 20 458 cells representing 9 previously reported subpopulations. We demonstrate that monocyte-derived macrophages closely mirror the transcriptomic signatures of the lipid-associated macrophage (LAM) in plaque, a population first discovered in liver and adipose tissue.^63,64^ A recent spatial transcriptomic study in the liver reported that LAMs are concentrated around portal veins, while the general monocyte/macrophage compartment is distributed across different zones.^77^ Although currently there is no spatial mapping of LAMs in human atherosclerotic plaques, it is reasonable to believe that plaque LAMs in vivo also originate from circulating monocytes.

With the high-resolution plaque macrophage atlas, we were able to further illustrate the transcriptomic heterogeneity within plaque LAMs and identify, for the first time, a CD52-hi subset expressing high levels of lipoprotein processing genes. Although direct quantitative measurement of lipid uptake in human plaque macrophages presents a challenge, the remarkable similarity in transcriptomic profiles between the lipid-handling macrophages and the CD52-hi LAM subset suggests a potentially significant role for these cells in in vivo lipid clearance. These findings may cast light on the rare adverse cardiovascular events including myocardial infarction that have been reported with anti-CD52 therapy (alemtuzumab).^78^ Binding of alemtuzumab to plaque LAMs within arterial walls may trigger intramural inflammation from acute damage to these cells and depletion of CD52-hi LAM from the vessel walls, thereby increasing CAD risk.

### FDX1/RDX Locus

*FDX1* encodes an important mitochondrial iron-sulfur protein reductase, which is an electron donor for many reducing reactions^79^ and is itself reduced by the NADPH-dependent ferredoxin reductase. Key mitochondrial complexes involved in respiration are regulated by FDX1-mediated lipoylation, and FDX1 deficiency results in loss of respiration.^80^ A genome-wide CRISPR screen demonstrated that it was essential for oxidative phosphorylation.^81^ While mitochondrial dysfunction is recognized as a contributory mechanism in the initiation of atherosclerosis,^82^ the role of ferredoxin has not been characterized in the context of coronary artery disease.

Our computational and experimental results show that the CAD risk T allele at rs10488763 in a distal enhancer region of the *FDX1* gene increases CAD risk by lowering *FDX1* expression in macrophages. The mechanism of this effect is likely complex. Specifically, we demonstrate the role of both AP-1 family factors and CEBPB in modulating the cis-regulatory effect of this CAD SNP. While the AP-1-binding motif is required for ox-LDL–induced enhancer activation, the expression of the AP-1 family member *FOS* negatively correlates with the expression of *FDX1* (Figure 2F). Overexpression of CEBPB bypasses the allelic effect of rs10488763 on enhancer activity and restores *FDX1* expression in macrophages with the disrupted AP-1-binding site. These results indicate a model in which AP-1 binding at the locus does not directly affect *FDX1* expression but rather remodels the local chromatin to create space for other TFs like CEBPB (Figure S9). This model is consistent with the observation that AP-1 factors bind collaboratively with cell-type–specific TFs to select enhancers and establish accessible chromatin.^83^

### Overview

Overall, we have developed a comprehensive framework that enables the systematic quantification of the disease relevance of heterogeneous macrophage subpopulations. This framework greatly facilitates the investigation of molecular mechanisms that account for the heterogeneity in the genetic risk conveyed by these subpopulations. Our work illustrates the substantial value of using single-cell information to improve the genomic resolution of the genetic architecture of human diseases.

## ARTICLE INFORMATION

### Acknowledgments

The authors thank all healthy volunteers who agreed to participate in this study. The authors also thank the Oxford Genomic Center for their support. Illustrations in Figure S1A, Figure S2D, and Figure S9 were created with BioRender.com.

### Author Contributions

C.A. O’Callaghan, T.K. Hiron, and J. Jiang contributed to conceptualization and design; T.K. Hiron, J. Jiang, T.A. Agbaedeng, Y. Malhotra, E. Drydale, J. Bancroft, and M.E. Reschen contributed to data collection; J. Jiang, T.K. Hiron, and C.A. O’Callaghan contributed to analysis and interpretation of results; C.A. O’Callaghan contributed to resources and funding acquisition and supervision. J. Jiang and C.A. O’Callaghan contributed to the manuscript drafting. All authors evaluated the results and commented on and approved the article.

### Sources of Funding

The research was supported by the Novo Nordisk Foundation (NNF15CC0018346 and NNF0064142), the British Hearth Foundation (RG/F/22/110085), the Wellcome Trust Core Award grant number 203141/Z/16/Z with funding from the National Institute for Health and Care Research (NIHR) Oxford Biomedical Research Centre. J. Jiang received funding from the China Scholarship Council (202006320024). T.A. Agbaedeng is supported by a Novo Nordisk Postdoctoral Fellowship run in partnership with the University of Oxford. The views expressed are those of the authors and not necessarily those of the National Health Service (NHS), the NIHR or the Department of Health.

### Disclosures

None.

### Supplemental Material

Expanded Materials and Methods

Supplemental Notes

Video S1

Tables S1–S6

Figures S1–S9

Major Resources Table

References ^12–14,18,23,38,49,51,53,54,56,60,61,65,69,84–98^
